# Characterizing the emergence and persistence of drug resistant mutations in HIV-1 subtype C infections using 454 ultra deep pyrosequencing

**DOI:** 10.1186/1471-2334-13-52

**Published:** 2013-01-30

**Authors:** Vijay Bansode, Grace P McCormack, Amelia C Crampin, Bagrey Ngwira, Ram K Shrestha, Neil French, Judith R Glynn, Simon A Travers

**Affiliations:** 1 Molecular Evolution and Systematics laboratory, Zoology, Ryan Institute, School of Natural Sciences, National University of Ireland Galway, Galway, Ireland; 2 Karonga Prevention Study, Chilumba, Malawi, South Africa; 3 Faculty of Epidemiology and Population Health, London School of Hygiene and Tropical Medicine, London, UK; 4 South African National Bioinformatics Institute, University of the Western, Cape, Bellville, South Africa; 5 Institute of Infection & Global Health, University of Liverpool, Liverpool, UK

**Keywords:** HIV-1, Drug resistance, Subtype C, Malawi, Ultradeep sequencing, Proviral DNA

## Abstract

**Background:**

The role of HIV-1 RNA in the emergence of resistance to antiretroviral therapies (ARTs) is well documented while less is known about the role of historical viruses stored in the proviral DNA. The primary focus of this work was to characterize the genetic diversity and evolution of HIV drug resistant variants in an individual’s provirus during antiretroviral therapy using next generation sequencing.

**Methods:**

Blood samples were collected prior to antiretroviral therapy exposure and during the course of treatment from five patients in whom drug resistance mutations had previously been identified using consensus sequencing. The spectrum of viral variants present in the provirus at each sampling time-point were characterized using 454 pyrosequencing from multiple combined PCR products. The prevalence of viral variants containing drug resistant mutations (DRMs) was characterized at each time-point.

**Results:**

Low abundance drug resistant viruses were identified in 14 of 15 sampling time-points from the five patients. In all individuals DRMs against current therapy were identified at one or more of the sampling time-points. In two of the five individuals studied these DRMs were present prior to treatment exposure and were present at high prevalence within the amplified and sequenced viral population. DRMs to drugs other than those being currently used were identified in four of the five individuals.

**Conclusion:**

The presence of DRMs in the provirus, regardless of their observed prevalence did not appear to have an effect on clinical outcomes in the short term suggesting that the drug resistant viral variants present in the proviral DNA do not appear to play a role in the short term in facilitating the emergence of drug resistance.

## Background

While the role of HIV-1 RNA in the emergence of resistance to highly active antiretroviral therapy (HAART) has been widely documented, less is known about the role of historical viruses stored in the proviral DNA. Following treatment interruption, latent viruses stored in the provirus have been shown to be responsible for the rapid rebound of viral load following treatment interruption [1-3]. However, Palmisano and colleagues suggested that the mutational archive stored in proviral DNA remains unchanged during HAART [4] with higher levels of DRMs observed in RNA extracted from circulating virus than those present in the provirus [5]. The suggested reason for these discrepancies is that standard bulk sequencing cannot fully access the spectrum of viral variants stored in the proviral DNA [5] where resistant viruses may be present in low abundance.

Recent research on HIV-1 in Karonga District, Malawi has shown that of the 40 HIV-1 subtype C infected individuals on HAART that we tested, 14% contained drug resistance mutations (DRMs) in the reverse transcriptase (RT) gene of viruses stored in the provirus [6] with clonal sequencing of latent viruses showing discrepancies in the presence and prevalence of DRMs in the proviral DNA [7]. These discrepancies further support the limitations of bulk sequencing for determining drug resistance in viruses contained within the proviral DNA. Traditionally, methods such as single genome amplification and real-time PCR have been used to identify the presence of low abundance viral variants [8,9]. We, and others, have shown the importance of clonal sequencing in revealing the spectrum of viral variants present within an individual [6]. However cloning and other traditionally used methods are labour intensive, expensive, time consuming and/or restricted to the detection of single variants. The advent of next generation sequencing platforms means that clinically relevant low abundance drug resistant HIV variants can be detected to prevalences as low as 1% of the viral population [10-16]. While several studies have found correlations between the presence of low abundance drug resistant viruses and clinical outcomes this has not always been the case [17,18]. To our knowledge, no studies have been undertaken to use ultra deep pyrosequencing (UDPS) to quantify the prevalence of low abundance drug resistant viral variants in the proviral DNA. Here, we undertake such a study and endeavor to correlate the presence/absence of such viruses with treatment outcome.

## Methods

### Sample selection

Samples were collected from five patients recruited to an antiretroviral cohort study between 2007 and 2009 from an ART clinic in northern Malawi. Antiretrovirals have been available in the District since 2005 and in this clinic since 2006. All patients have been started on a fixed dose combination of stavudine, lamivudine and nevirapine on the basis of clinical staging or CD4 count. Previous work identified discrepancies or ambiguities in the presence/absence of drug resistant mutations in viral sequences from samples collected from these five individuals at sequential time-points during treatment [6]. Fifteen samples representing an average of three sampling time-points from five of these patients were selected for subsequent analysis using ultra-deep pyrosequencing in this current study (Table 1).

**Table 1:** Samples subjected to ultra-deep pyrosequencing in this study

**Patient Number**	**Sampling Time-point**	**NRTI mutations**	**NNRTI mutations**	**Primary PCR**	**Secondary PCR**
**Patient 2**	**Baseline**			9	9
	8 Months		**K103KN**		
	**11 Months**		**Y181C**	10	20
	**14 Months**			3	20
					
**Patient 32**	**Baseline**		V90I	6	9
	**7 Months**	T215ST		2	20
	8 Months	**M184I**			
	14 Months				
	15 Months		**G190AE**		
	**16 Months**		V108AV	3	20
	**24 Months**			2	20
					
**Patient 42**	**Baseline 1 (2008)**			2	7
	**Baseline 2 (2009)**	V118IV		5	20
	5 Months	V118I			
					
**Patient 45**	**Baseline**		V106I, E138A, **G190A**	9	20
	**8 Months**		V106I, E138A, **G190A**	10	20
	**13 Months**		Sequence Failed	10	20
					
**Patient 76**	**Baseline**		V90I, **Y181CY**, H221HY	10	20
	3 Months				
	**6 Months**		**Y181C**	10	15
	**9 Months**		Sequence Failed	10	19

Consensus sequencing of reverse transcriptase from five patients had previously revealed discrepancies in the presence/absence of DRMs [6,7]. Baseline describes the sampling time-point prior to ART initiation with subsequent samples being described as the number of months post ART initiation. Mutations shown in the table are those observed in the consensus sequencing of each sampling time-point with DRMs to current therapy marked in bold. The sample time-point names for samples selected for subsequent ultra-deep pyrosequencing are marked in bold and the success rate for multiple PCRs for these samples are also shown. The remaining six samples were not chosen due to difficulty/failure with PCR amplification or consensus sequencing.

### Ultra deep pyrosequencing (UDPS)

DNA extraction and nested PCR was performed as previously described [6] with the second round PCR primers modified to include the A and B adaptors necessary for 454 sequencing. Each second round primer also included a unique sequence tag (MID) to enable us to distinguish between samples from the multiplexed sequencing reaction. The region targeted for amplification was 760 nucleotides in length covering amino acid positions 13 through 257 of reverse transcriptase (HXB2 numbering). Temperature gradient PCRs identified 57.5°C as the optimum annealing temperature for all secondary PCR reactions with the extended primer sequences for UDPS. Considerable effort was invested into optimizing the PCR amplification approach to account for potential over-amplification of a subset of viral variants present in the quasispecies. For each sample the final PCR strategy involved performing ten first round 100 μl PCR reactions. 5 μl of the product of each primary PCR was used as starting material for two separate secondary PCR reactions, generating a maximum of 20 secondary amplification reactions from each sample. PCR products were quantified on 1% agarose gels in comparison with the Hyperladder 1 molecular marker (Bioline). The 20 PCR products generated from each DNA sample were then mixed in equimolar amounts and an aliquot of the mixed products arising from each DNA sample were then electrophoresed side by side for comparison and further confirmation of quality and quantity. PCR products from all samples were then further pooled in equimolar amounts. Amplicon sequencing was performed on one half of a picotiter plate using a 454 Genome Sequencer FLX by LGC Genomics (Germany).

### Data cleaning and analysis

Sequence reads from each individual sample were separated based on their MIDs and subsequent quality control and analysis was performed independently for the reads corresponding to each sample. Next, the data were trimmed using the modified-Mott algorithm implemented in Geneious 5.4.3 [19] with a 0.01 error probability limit. Initially the identity of the resulting reads to their respective consensus sequence was determined using the BLAST-like word (k-mer) matching approach implemented in Segminator 1.3.2 [20] with a word size of five and a read quality of two. Reads with significant identity were aligned to the consensus sequence excluding any reads less than 22 nucleotides in length, the dataset was translated to amino acids and the frequency of amino acids at each position was determined using Segminator 1.3.2 [20].

The prevalence of drug resistant mutations (DRMs) was determined for each sample with three categories of DRMS identified. Firstly, DRMs detected in less than 1% of the reads sequenced from the amplified viral population for that position were discounted to account for potential errors due to the error rate of PCR and UDPS. The second category of mutations consisted of those with a prevalence greater than 1% and less than 20%, corresponding to those that cannot be determined using consensus sequencing. The final category consisted of those DRMs with prevalence greater than 20%, comprising mutations that can, in theory, be observed using traditional consensus sequence genotyping. Given that the number of reads that were sequenced at each timepoint in a patient were unlikely to be exactly the same, we implemented a statistical approach for prevalence calculations as opposed to using a rigid fixed cutoff. For every drug resistant mutation, the 95% confidence interval from the binomial sampling distribution was calculated for the observed frequency taking into account the number of reads sequenced at that timepoint. The upper 95% confidence interval was used as a cutoff for the allocation of DRMs into both the greater than 1% and less than 20% category and the greater than 20% category.

Recent work has described HIV drug resistance mutations that are located within, or adjacent to, homopolymers in the HIV Pol gene [21]. Given that one of the known shortcomings of the 454 pyrosequencing approach is that it can cause false insertions or deletions in homopolymeric tracts, we undertook an approach to verify if drug resistance mutations at such homopolymers were genuine DRMs or arose as the result of sequencing error. We have developed a novel tool called rapid amplicon mapping in codon space (RAMICS) that uses hidden markov models to map next generation sequencing reads to a reference sequence in codon space, thus generating a biologically relevant alignment (Wright et al., in prep). This approach identifies the presence of hompolymer runs in data and considers them in the mapping process, thus accounting for the possible variances in length as a result of sequencing error. Using RAMICS we confirmed whether resistance calls at DRMs located at homopolymer tracts in the sequence data for all of the datasets were biologically relevant or had arisen as a result of sequencing error.

Permission for the study was received from the National Health Sciences Research Committee, Malawi, and the Ethics Committee of the London School of Hygiene and Tropical Medicine, UK. Written informed consent for participation in the study was obtained from all participants.

## Results

### DNA amplification and UDPS data cleaning

Despite considerable effort, PCR amplification was not uniformly successful for all DNA samples. Secondary PCR was successful from ten primary PCRs for six of the 15 samples with all 20 secondary PCR reactions being successful in four of these (Table 1). Amplification from primary PCRs was less successful for the remaining samples ranging from positive secondary amplification from two to nine first round reactions (Table 1). In instances where secondary PCRs were unsuccessful from primary reactions, additional secondary PCRs were performed from those primary PCR products that did yield amplification, in order to maximize diversity (Table 1). Despite multiple attempts to obtain 20 secondary PCR amplifications, fewer than 10 secondary products were obtained from the baseline sample from patients 2, 32 and 42. Although secondary amplification was successful in all 20 reactions from the 2^nd^ baseline sample from patient 42, the amplification was very weak compared to secondary amplifications from other patients. Similar results were obtained from 16^th^ month from Patient 32 and 13^th^ month sample from patient 45.

### Deep sequencing and data cleaning

454 sequencing resulted in a total of 372,169 reads of which 1038 could not be sorted on the basis of their MID. Of the remaining 371,131 reads the number of sequences representing each of the 15 samples ranged from 12,926 to 38,514 (Additional file 1: Figure S1A). Quality trimming was employed with a 0.01 error probability limit meaning that each trimmed read has a minimum average accuracy greater than 99%. This trimming step did not result in the removal of any reads (as no minimum length requirement was imposed in this step), however the average length of all reads was significantly decreased from 541 for reads before trimming to 176 for reads following trimming (Additional file 1: Figure S1B).

The number of quality trimmed reads we attempted to map to the template sequence ranged from 12,925 to 38,459 with reads being excluded from the mapping procedure as a result of falling below the minimum read length score (22 nucleotides) or due to low identity to the template sequence. Between 677 and 3007 reads per patient were excluded based upon the read length cutoff (Additional file 1: Figure S1B) with a range of 1324 and 14,609 reads per patient excluded because of low identity to the template. Three samples (Patient 45 at 13 months, patient 32 at 16 months and patient 42 baseline 2) had large numbers of reads excluded during the mapping process with 43%, 61% and 65% percent of reads removed respectively. In each of these cases the majority of reads were removed due to low identity to the template sequence. Further analysis of the excluded reads from these individuals showed that a large proportion of them mapped to regions throughout the HIV genome outside of the region of interest. Sequencing coverage towards the centre of each amplicon was significantly less than at either the 5’ or 3’ end of the amplicon.

### Prevalence of drug resistance mutations identified with deep sequencing

**Figure 1:**
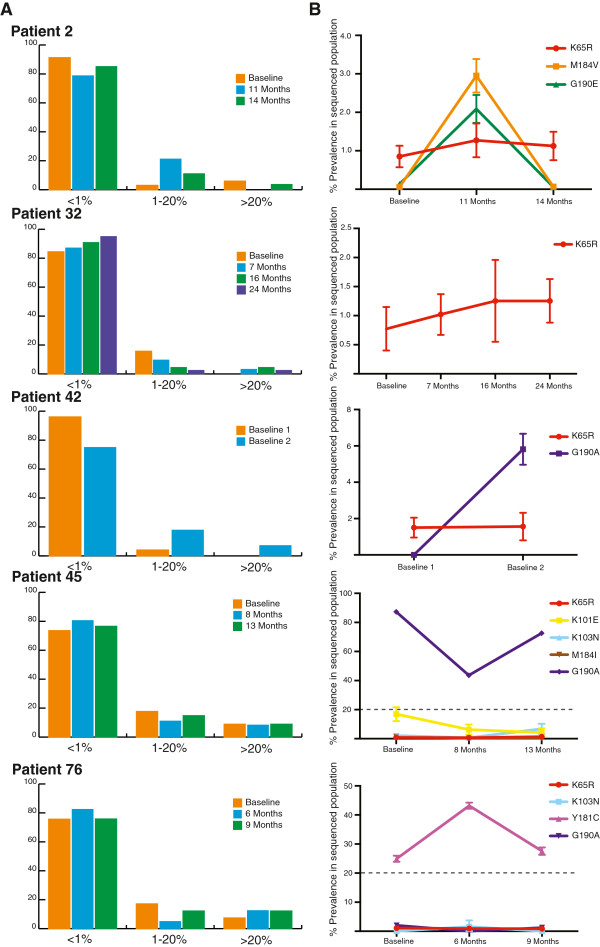
**Observed DRMs at all sequenced sampling time-points for each patient.** Panel **A** shows the percentage of DRMs expressed as in each prevalence category for each patient. DRMs observed at less than 1% of the amplified and sequenced viral population were discounted from further analysis in this study to account for potential errors due to the error rate of PCR and UDPS but are shown in this figure for comparison purposes. Panel **B** shows the prevalence of DRMs present in the amplified and sequenced viral population against the patients’ current therapy at each of the sampling time-points. For each prevalence point, the upper and lower 95% confidence intervals from the binomial sampling distribution are shown as error bars. DRMs with a prevalence less than 1% of the amplified and sequenced viral population are not included.

In all samples the vast majority (between 74% and 96%) of drug resistance mutations identified by deep sequencing were detected at prevalence levels less than 1% of the sequenced viral population (Figure 1A) and were, thus, excluded from any subsequent analysis. For the remaining DRMs, we assessed the effect of sequencing error at homopolymeric regions and, in all instances, we found that sequencing error was not responsible for the resistance calls indicating that they are, in fact, genuine. An average of 11% of observed DRMs (range 2.5-21.21%) were detected at prevalence levels between 1% and 20% of the sequenced viral population with an average of 6% (range 0–12.5%) identified at greater than 20% prevalence in the sequenced viral population (Figure 1A and Table 2).

**Table 2:** Prevalence of DRMs in pyrosequencing data

**Patient number**	**Sampling time-point**	**CD4 count**	**1-20% prevalence**	**>20% Prevalence**
**Patient 2**	Baseline	64	**K65R**	
	11 Months	N/A (9 months: 110)	**K65R**, **M184V**, **G190E**	
	14 Months	153	**K65R**	
**Patient 32**	Baseline	271	**K65R**, V90I	
	7 Months	384	**K65R**	V90I
	16 Months	N/A (19 months: 443)	**K65R**	
	24 Months	N/A	**K65R**	
**Patient 42**	Baseline 1 (2008)	N/A	**K65R**	
	Baseline 2 (2009)	362	**K65R**, A98S, **G190A**	
**Patient 45**	Baseline	55	**K65R**, A98S, **K101E**, K103E, **K103N**, K103R, **M184I**	V106I, E138A, **G190A**
	8 Months	216	**K65R**, A98S, **K101E**, K103E, K103R	V106I, **G190A**
	13 Months	258	**K65R**, **K101E**, K103E, **K103N**, K103R	V106I, E138A, **G190A**
**Patient 76**	Baseline	118	**K65R**, T69I, **G190A**, H221Y	V90I, A98S, **Y181C**
	6 Months	N/A (8 months: 269)	**K65R**, **K103N**, H221Y	V90I, A98S, K103E, **Y181C**, H221Y
	9 Months	N/A (12 months: 268)	**K65R**, V90I, K103E, **G190A**, H221Y	A98S, K103R, **Y181C**

Only DRMs observed at >1% in the sequence data are shown. DRMs relevant to each patient’s current therapy regime are shown in bold and those that were also seen in consensus sequencing are underlined. CD4 counts (x10^-6^/L) are shown. When a CD4 count was not available for a sampling time-point the count from the sample taken at a date closest to that time-point is shown.

UDPS identified an additional 32 DRMs (17 against current therapy) that had not been observed by consensus sequencing (Table 2). The vast majority of these (26) were observed at a prevalence between 1-20% of the sequenced viral population (Table 2). Bulk consensus sequencing was not successful for the final samples from patients 45 and 76 (Table 1), however UDPS identified DRMs at both 1-20% (9 DRMs, 4 against current therapy) and greater than 20% prevalence (6 mutations, 2 against current therapy) within the sequenced viral population (Table 2). Of the 15 DRMs (five to current therapy) seen in bulk sequencing of samples subjected to UDPS (Table 1), 10 of these (four against current therapy) were also observed in the UDPS sequencing with the vast majority of these (8 of the 10) being seen at a prevalence greater than 20% in the sequenced viral population (Table 2). The two DRMs observed at less than 20% of the sequenced viral population were V90I (9.5% prevalence) and H221Y (7.7% prevalence) in the baseline samples from patients 32 and 76 respectively. Three DRMs identified in bulk sequencing (Y181C in patient 2 at 11 months and T215ST and V108AV in patient 32 at 7 and 16 months respectively) were not observed at any level of prevalence in UDPS. DRMs V118I and E138A, from patients 42 (baseline 2) and 45 (8 months) respectively, were observed in the UDPS data but sequence coverage at these positions was below the cutoff.

Of the mutations detected at greater than 1% prevalence by UDPS against current 1^st^ line therapy, only K65R was observed, albeit at varying levels, in all of the patients studied (Figure 1). In all patients the prevalence of K65R in the sequenced viral population was observed at less than 20%, however the prevalence in each individual increased over time. Only in two patients were DRMs to current 1^st^ line therapy observed at prevalence greater than 20% of the sequenced viral population with G190A and Y181C seen in patients 45 and 76 respectively (Figure 1). In both of these cases the DRM was already present in the patient’s viral population prior to antiretroviral (ARV) exposure and was observed to fluctuate during exposure (Figure 1). A similar fluctuating pattern was seen in patient 2 whereby M184V and G190E were not identified by UDPS at baseline or at 14 months yet represented 3% and 2% of the sequenced viral population respectively in the sample at 11 months (Figure 1). Aside from DRMs against current 1^st^ line therapy 13 DRMS against other ARVs were identified in four patients (Additional file 2: Figure S2). These mutations were mostly polymorphisms that have limited effect on drug resistance.

## Discussion

We have used ultra deep pyrosequencing (UDPS) to investigate the presence of low abundance drug resistance mutations present within the proviral DNA of individuals on ART. Previous studies have suggested that the mutational archive stored in proviral DNA remains unchanged during HAART [4] with higher levels of DRMs observed in RNA extracted from circulating virus than those present in the provirus [5]. The suggested reasons for these observations are that standard bulk sequencing cannot fully access the spectrum of viral variants stored in the proviral DNA [5] coupled with the fact that resistant variants may be present at low prevalence within the proviral DNA. The sensitivity of UDPS to detect drug resistant minor variants is limited by the number of virus templates that can be successfully extracted and amplified using PCR [12,22]. Through our thorough PCR approach coupled with the use of UDPS as opposed to consensus sequencing we sought to maximise the genetic heterogeneity that was amplified and subsequently sequenced from the proviral DNA. As might be expected, there was an association between PCR amplification success and sequencing success. For patient 32 month 16, patient 45 month 13, and the 2^nd^ baseline sample from patient 42, PCR amplification was weaker and thus a higher volume of PCR amplicon had to be added to reach the same final quantity for sequencing as for other samples. For these three samples far more sequences resulting from pyrosequencing had low identity with the consensus sequence and were excluded. It was still worth carrying out pyrosequencing on these samples, however, as thousands of sequences with high identity were returned for analysis. The differences in numbers of quality reads between samples would prevent comparisons of proportions of DRMS across these samples, but not the identification of minor variants.

Bulk sequencing approaches can only detect viral variants present in greater than 20% of the viral population [23-25] and we did see strong correlation between DRMs observed in bulk sequencing and those at greater than 20% of the sequenced viral population in the UDPS at the same time-point. There were, however, further DRMs observed at greater than 20% prevalence that had not been identified using bulk sequencing and the observation of these are likely the result of our approach successfully accessing a greater level of the viral diversity present in the proviral DNA. We would have expected UDPS to detect all of the mutations that were detected via bulk sequencing but this was not the case. From each of five patient samples one DRM, retrieved using the bulk sequencing approach, was absent from the reads retrieved from the samples through the UDPS approach. The phylogenetic origin of each sequence from the bulk sequencing approach was confirmed as belonging to the individual in question, as was the presence of the DRM in the sequence chromatograph indicating that the reporting of the original mutation was not an error, nor was there a mix up of samples. For two samples (patient 32 sample 16 month and baseline 2 samples from patient 42) poor PCR amplification success may be responsible for some mutations being absent. However, for the other three samples this is not the case. Despite utilizing multiple primary and secondary PCRs to maximize the diversity sampled, PCR bottleneck still cannot be excluded as a cause of the absent DRMs. However, as PCR amplifications were from proviral DNA it is perhaps not surprising that some differences would be found between amplicons deriving from different aliquots of DNA, as latent viruses may be more diverse than circulating virus. It should be noted that the presentation of prevalence in our work, as with all other similar studies, should be interpreted as the prevalence observed in the amplified and sequenced viral population as opposed to being a direct measure of the prevalence of a variant in an individual’s viral population. The discrepancy between these two interpretations has yet to be elucidated however the exciting development of degenerative primer ID approaches [26] will enable such quantification of these potential biases in the near future and help further resolve the PCR bottle neck issue.

Resistant viruses that make up as little as 1% of the viral population within an individual have been suggested to be clinically important as they can expand rapidly under the selective pressure exerted by exposure to HAART [10-16]. The ability of UDPS to effectively quantify such variants is limited by PCR and sequencing induced errors. Hedskog and colleagues eloquently showed that the error rate of UDPS is not uniform across sites within the pol gene and that PCR-induced recombination is minimal [27]. Learning from reported issues in other studies we used larger sample volumes [28-30], a high depth of coverage [12,28-30] and a somewhat conservative cutoff of 1% for identifying low prevalence variants. In all patients we identified low abundance variants with DRMs against the current 1^st^ line therapy. In particular K65R, strongly associated with the development of virologic failure in subtype C infected individuals [31-37], was observed at low abundance in all patients. While we cannot rule out that the observation of low abundance K65R at various time-points in all patients is not as a result of the previously reported propensity for PCR error at this position in subtype C viruses [38] we did use a high fidelity PCR enzyme and multiple primary PCRs to try to avoid such PCR error.

Despite the observation of low abundance drug resistant variants in all individuals, these viruses appear to have had minimal effect on measured treatment outcome. Due to the geographically isolated setting resulting in logistical difficulties viral loads are not routinely assessed and treatment success, to date, has been measured using WHO stages and CD4 counts. Thus, at the final sampling timepoint one (patient 2) is defined as having immune failure (CD4 cell count <200 cells/mm^3^ after at least 12 months on ART) with all remaining patients showing satisfactory treatment response. While the CD4 counts of two of these individuals, patients 45 and 76, were sufficiently high to avoid being interpreted as treatment failures, their semi-borderline CD4 counts (258 and 268 CD4 T cells cells/mm^3^) coupled with the presence of multiple minor variant DRMs in their provirus could suggest that these patients may be at high risk for imminent failure. Nevertheless, the CD4 counts of all patients rose over the course of the study despite the detection of low abundance variants at early timepoints and all of the participants remained on 1^st^ line therapy until their final sampling timepoint up to as much as 24 months after treatment initiation. Thus, it appears that the presence of low abundance DRMs in the provirus of these individuals has little effect on treatment outcome in the short-term. Previous work suggests that HIV rebounds from latently infected cells rather than as a result of continuing low-level replication [1] and, thus, there is always a possibility that these low abundance resistant variants could emerge to dominate from the proviral DNA following treatment interruption or as a result of poor adherence.

## Conclusion

We observe, in the individuals on continuous antiretroviral therapy studied here, low abundance drug resistant viral variants present in the proviral DNA do not appear to play an immediate role in facilitating the emergence of drug resistance through emergence to dominance. Whether this is true in all instances should be explored further in future longitudinal studies and this group of patients should be monitored further given that in all of the individuals we detected minor variants with DRMs against their current treatment regimen.

## Competing interests

The authors declare that they have no competing interests.

## Authors’ contributions

VB performed the amplification and sequencing of samples and wrote the first draft of the paper. AC, BN, NF and JRG were responsible for the management and running of the Karonga Prevention Study ART project, the collection of samples and all clinical aspects of the project. RKS undertook the analysis examining the effect of sequencing error at homopolymer regions on the calling of drug resistance mutations. GPM supervised the wet-lab work and the interpretation of results while SAT undertook the preliminary bioinformatics analysis of the sequence data and authored subsequent drafts of the paper. All authors contributed to the final draft.

## Pre-publication history

The pre-publication history for this paper can be accessed here:

http://www.biomedcentral.com/1471-2334/13/52/prepub

## Supplementary Material

Additional file 1: Figure S1 454 sequence quality control. (**A**) The total number of sequenced reads obtained for each sampling time-point is shown as the total size of the bar representing each time-point. The number of reads mapped to the reference sequence and removed as a result of length and identity cutoffs are also shown. (**B**) The mean and range of read lengths observed for each sequenced sample are shown both before and after quality trimming. In all cases the mean and range of read lengths decreases significantly following trimming. Click here for file

Additional file 2: Figure S2 Observed DRMs against ARVs not present in the patients’ current regimen at all sequenced sampling time-points for each patient. DRMs with observed prevalence less than 1% of the amplified and sequenced viral population are not included. For each prevalence point, the upper and lower 95% confidence intervals from the binomial sampling distribution are shown as error bars. Click here for file
